# Parental and healthcare provider attitudes towards the Healthy Child Programme in England: a qualitative analysis

**DOI:** 10.1186/s12889-024-19515-5

**Published:** 2024-08-28

**Authors:** Tahmid Rahman, Joseph Freer, Isabella Cordani, Michael Papasavva, Leo Dunkel, Robert Walton, Helen L. Storr, Andrew J. Prendergast, Joanna Orr

**Affiliations:** 1 Royal Holloway University of London, London, UK; 2 Queen Mary University of London, London, UK

**Keywords:** Health visiting, Child development and growth, Service empowerment, Healthcare accessibility, Preventative care, Tower Hamlets

## Abstract

**Background:**

The Healthy Child Programme (HCP) in England, delivered by Health Visitors (HV) and Nursery Nurses (NN), aims to assess growth and development in pre-school age children. This qualitative analysis aimed to evaluate the perceptions and experiences of HCP providers and parents located in a London borough.

**Methods:**

This qualitative analysis is part of a larger study piloting an automated growth screening algorithm in a London borough. We conducted three focus group discussions; two with parents of pre-school children participating in the pilot study, one in English (*n* = 6) and one in Sylheti (*n* = 5), and one with HVs and NNs (*n* = 11). Sampling was purposeful, and written informed consent was obtained. Groups were facilitated by the same bilingual researcher using semi-structured topic guides. Data were analysed using reflexive thematic analysis and assessed for intercoder reliability.

**Results:**

Three broad themes were identified in the data: (1) lack of clarity around the role of the HV and NN; (2) a lack of resources; and (3) a desire for a preventative service. Underlying these themes was a sense of disempowerment shown by HVs/NNs and parents, as well as systemic issues in terms of the accessibility and practicality of the service. Nevertheless, parents and HVs/NNs all stressed the importance of the service in providing information, reassurance and advice.

**Conclusions:**

Various challenges prevent the HCP from providing equitable and effective care to every child. However, the service was recognised as very valuable by users and providers despite systemic difficulties.

**Supplementary Information:**

The online version contains supplementary material available at 10.1186/s12889-024-19515-5.

## Background

The Health Visiting service in England comprises a cadre of nurses and midwives with specialised training, who deliver public health services to mothers, families, and communities. These Health Visitors (HVs) deliver the Healthy Child Programme (HCP) which focuses on child health and development from ages zero to five years. The HCP offers five mandated contacts, from pregnancy to 2 years, to assess the overall child health and development, as well as providing screening, immunisations, advice to parents and signposting to other services. HVs are supported by, and collaborate with, a wide range of other NHS services and teams, including paediatricians, GPs and Nursery Nurses (NNs). HVs and NNs deliver many of the mandated HCP visits, with NNs conducting most 2-year assessments. The HCP offers universal and targeted levels of service depending on the needs of individual families [1]. Similar well child or child health surveillance programmes exist in the United States, Australia, France, the Netherlands, Sweden and other high-income settings and although there are structural differences the basic elements of the programmes are consistent [2]. 

While no formal evaluation of the HCP has been undertaken, each of the programme’s areas of intervention is grounded in strong evidence of positive impacts on maternal and child health [3–5]. The HCP seeks to address many maternal and child health outcomes, making evaluation of the whole programme challenging. Parental views and attitudes towards the health visiting service have been shown to be positive when they have a good relationship with their HV/NN, when they feel the HV/NN is knowledgeable and listens to their concerns. On the other hand, negative experiences of the service emerge when there is tension in the HV/NN-parent relationship [6, 7]. Migrant parents may experience health services for their children differently and their expectations may be shaped by previous experiences in their countries of origin [8]. There is a lack of evidence focused on the HV/NN perspective, and whether parental and HV/NN perspectives align.

The UK National Health Service (NHS) has experienced funding cuts in recent years, resulting in systemic problems such as staff shortages, difficulty recruiting, long waiting lists for onwards referrals and difficulties arranging appointments [9–12]. Cuts to interventions that promote child health have impacts on current and future population health, as well as deepening health and social inequalities [13, 14]. Drastic declines in health outcomes such as infant mortality, obesity and tooth decay in pre-school aged children are areas where the health visiting service is well positioned to have a strong positive impact when resources are in place [15].

### Research Aims

To observe and interpret the range of factors involved in shaping HV/NN visits, this sub-study posed the following questions:

1. How do parents and HV/NNs describe health visits and the role of a HV/NN?
2. How do parents and HV/NNs envisage service development?
3. What are the challenges and limitations to service development?

## Methodology

### Study design

This qualitative study was conducted as part of a larger pilot project, the Child Development and Growth in East London study (CDGEL). CDGEL was based in a borough of east London with a high level of deprivation. The study sought to generate quantitative and qualitative data on the feasibility and acceptability of a proposed growth screening programme, implemented at age 2 years through HV/NNs. The current analysis utilizes data from a qualitative sub-study among parents and healthcare providers, to evaluate perceptions and experiences of the HCP. We explored how this programme works in east London and identified areas where delivery could be strengthened.

### Reflexive positioning

Two researchers, TR and JO, led on data collection and analysis. TR is a male PhD candidate with experience in qualitative methods. His research interests include community wellbeing and the social production of mental health. JO is a female postdoctoral researcher with experience in mixed methods research. Her research interests include child growth and the social determinants of health.

### Participants

As part of CDGEL, focus groups were conducted with parents and HV/NNs to evaluate attitudes towards growth screening. Parents were purposively sampled, with every caregiver who attended a study visit within the recruitment timeframe being invited to participate. We aimed to recruit ten participants per focus group, and invitations were halted once a suitable number of participants confirmed attendance. One group was conducted in English and one in Sylheti. HV/NNs were also purposively sampled, with an email invitation circulated to every HV/NN and nursery nurse in the London borough where the study took place and recruitment again halted once a suitable number of participants had agreed to participate. To be eligible, HV/NNs were required to be currently working within the Healthy Child Programme in the area.

### Data collection

Two semi-structured topic guides were prepared by JO to facilitate data collection and ensure the research questions were addressed (see appendix 1 and 2). Every group was facilitated by the same English and Sylheti-speaking bilingual researcher, TR, who also translated the parent data collection instrument into Sylheti. The HV/NN focus group was conducted online via Zoom, while the parent focus groups were conducted in person. This reflected the schedules and availability of each group. All sessions were audio recorded, and data transcribed by TR. TR and JO were present at each session and both kept field notes. For in-person focus groups, childcare was provided through a mobile creche. Participants were provided with participant information sheets in advance and were given the opportunity to speak to the study team prior to the sessions. Three of the participants in the HV/NN group were part of the study team who collected data for the wider pilot study. As such they were familiar with the researchers and the background and purpose of the study. There were no other prior relationships between participants and study team. Transcripts were not shared with participants for further feedback. Informed written consent was obtained from each participant.

### Data analysis

To recognise the multimodal construction of health visits, poststructural realism (Heller, 2008) provides an apt approach to epistemology and ontology; Heller affirms that meaning is socially constructed but that it is also possible to ascertain a sense of reality through reference to material resources alongside social relations. In turn, both interpretations of interview data and indications towards related material conditions are taken as subjects of study. The analysis was conducted through reflexive thematic analysis (RTA) [16], to suit the theoretical flexibility required to interpret interview data. We also drew on discourse analytical modes, positioning analysis, and systemic thinking that ties the data to its social and material contexts. Notably, regarding access to services, in positioning theory (Lefebvre, 1991; Moore, 2009), ‘centres’ describe the top of a hierarchy, where power, wealth, information and more are located, while ‘peripheries’ are distanced from these centres. Positioning theory is drawn upon to identify that participants can feel as though they are distanced from the social position of those who would be empowered by the NHS. Where appropriate in the analysis, further analytical modes are used and explained in keeping with the theoretical flexibility of RTA.

We did not seek to achieve data saturation; data saturation is conceptually incompatible with RTA due to the ongoing data interpretation process [17]. The concept of information power is better suited to evaluate the appropriateness of the sample size [18]; the sample were broad and diverse enough to capture data on the topic of parental and healthcare professional experiences of the HCP. As the data were initially collected as part of a wider project with a specific aim, sample size calculations were not conducted for the current analysis.

Analyses were conducted by TR and JO. In using RTA, TR and JO familiarised themselves with the dataset by writing reflexive post-session notes and reading through before coding. Initial themes were discussed around the research questions which involved reflections on inductive, data-driven observations alongside theory and research-driven deductions.

### Ethics

Ethical approval for this study was obtained from the Wales Research Ethics Committee 4 (21/WA/0385).

## Results

A total of 21 participants took part (11 parents and 10 healthcare professionals) in the focus groups. Further participant characteristics are outlined in Table 1. Three overarching themes, subthemes and a summary of each are presented in Table 2. In the text, quotes are attributed to parents (‘P’), or HV/NNs (‘HVNN’).

**Table 1:** Participant and interview characteristics

Group	Format	Duration	Language	Participants	Inclusion and exclusion criteria
HV/NNs	Online (Video Call)	90 min	English	7 HVs (1 male, 6 female) 3 NNs (3 female) (10 total)	Inclusion: HV/NN Working in HCP Working in the London borough where the study was based Exclusion: None
English language parents	In-person	60 min	English	6 mothers	Inclusion: Parent of study child Living in the London borough where the study was based Comfortable speaking in English Exclusion: None
Sylheti language parents	In-person	50 min	Sylheti	4 mothers 1 father (5 total)	Inclusion: Parent of study child Living in the London borough where the study was based Comfortable speaking in Sylheti Exclusion: None

**Table 2:** Data themes and sub-themes

Themes	Subthemes	Summary
1. **The role of the HV/NN and the health visiting service**	• Lack of clarity around the healthcare system • Navigating the system	Parents and HV/NNs both described health visits as meetings in which parents seek support from experts concerning the growth and development of their child. However, both parents and HV/NNs relayed that parents are unclear about the role of HV/NNs, the extent of support they can provide, and how to navigate wider systems of healthcare.
2. **Strained staffing and resources**	• The impact of the pandemic • Inflexibility • Lack of continuity of care results in parents receiving mixed advice • Power to refer for health and social issues	Limitations on the service provided by HV/NNs were frequently linked to changes in resourcing alongside wider social issues after the COVID-19 pandemic. Many HV/NNs relayed that difficulties with the job are not specific to HV/NNs: “the challenges are just with anything in the NHS: staffing, resources, but we do our best” (HV 1). The sustained disempowerment of health visiting programmes, HV/NNs, and parents, led to much of the hope for improvements revolving around empowering the service to challenge some of the inefficiencies in the NHS while providing staffing and resources where they are most needed.
3. **Desiring a preventative model**		Health visits were imagined as part of a preventative and holistic model of healthcare that is attentive at its earliest stages to the varying needs a child may have around growth and development, with a focus on preventative care.

### Theme 1: the role of the HV/NN and the health visiting service

#### Subtheme 1.1: lack of clarity around the healthcare system

Parents reported difficulties navigating the healthcare system when they need support for their children, and confusion around the health visiting service compounded that lack of clarity. This was expressed in both parent groups. Many migrant parents felt that their unfamiliarity with the system made it more difficult to navigate, but British parents also validated the difficulties of the system:

*Especially if you haven’t grown up in this country […]*,* it’s really difficult to know [what support children will receive].*

P1, Mother, English Language Focus Group.

*I grew up in this country*,* and I didn’t know any of this either. I think most of the information I got about services were through either other mums or through children’s centres […] I think that’s where I got most of my information.*

P2, Mother, English Language Focus Group.

This need to actively seek support was repeated by parents who reported only having reviews “*because I called and I was chasing*” (P3, Mother, English Language Focus Group). Moreover, when other services were involved, the lack of clarity in the system meant that some parents were sent back and forth between services. HV/NNs in turn echoed the same frustrations in working with other parts of the healthcare system:

*[HV/NN] said*,* “oh*,* he’s delayed. What do I do next? Where do I have to go? What shall I do?” They didn’t say anything. They say*,* “oh*,* you need to inform your school”? When I say it in the school*,* they say “you’re supposed to inform your health visitor”? I don’t know what to do. When I speak to one*,* they say go to the other.*

P4, Mother, English Language Focus Group.

*Nine times out of 10 some of the GPs refer the families back to us. It turns into a game where: “No*,* go and see a GP. No go and see a health visitor.” And the families are like*,* “Well who do I see?” So*,* it does become a bit of a frustration there.*

HVNN1, Nursery Nurse.

When describing the role of HV/NNs, parents reported being confused about what support the HV/NNs could provide and connected this to the HV/NN’s seemingly limited ability to act upon what they observed despite having expertise:

*What happens if there is a problem? […] Because that’s where I’m still trying to understand well*,* what is the health visitor’s role in this? […] is it to connect you with a specialist? To give you a referral? […] How do we use that resource?*

P4, Mother, English Language Focus Group.

Despite most of the participants having acknowledged that HV/NNs “*can give advice about what’s concerning*,* and they can educate us about it*” (P5, Mother, Sylheti Language Focus Group), health visits were compared against specialist interventions and secondary care; parents were unclear as to whether health visits are a form of intervention or assessment. This uncertainty also produced hesitancy towards health visits.

*I found this health visitor concept to be a bit weird because I had the impression it’s much more about checking on parents*,* whether they’re doing their job rather than actually checking the health of the kids.*

(P6, Mother, English Language Focus Group)

The HV/NNs echoed similar sentiments showing awareness that some parents did not trust the service. The need for parents to be receptive to information was stressed in recognition that a good health visit is collaborative:

*The expectations from the families […] is a quick fix and it’s not like that. It’s not like […] you see the health visitor*,* and everything is okay. It involves parents’ and health professionals’ interaction.*

(HVNN2, Health Visitor)

#### Subtheme 1.2: navigating the system

This situation has led some not to seek support from HV/NNs in pursuit of a primary care service that would have been more empowered to help them:

*I think I’ve been to the A&E more times than I’ve seen a health visitor […] And maybe that’s also because I just don’t know the system and I don’t even know where to call.*

P4, Mother, English Language Focus Group.

Despite this lack of clarity, there remained a recognition that health visits can be beneficial, primarily as a source of health education and to provide reassurance for parents:

*I feel like*,* in that profession*,* they are the experts so I trust them. They studied to be in the profession*,* so you have to trust them to take care of us.*

P5, Mother, Sylheti Language Focus Group.

This recognition of professional judgement and the value of human intervention was drawn on as a major strength of the health visits by HV/NNs who emphasised this value within a strained healthcare system:

*Because the resources are so limited*,* it’s more important than ever that health visitors are delivering the Healthy Child Programme to try and impact the behaviour of families.*

HVNN3, Health Visitor.

### Theme 2: strained staffing and resources

#### Subtheme 2.1: the impact of the pandemic

The COVID-19 pandemic was reported to have had direct and indirect impacts on health visits.

Parents and HV/NNs agreed in their perception of a health service that has become less personal and seemingly engages more superficially:

*I had a very different experience for both children so for the eldest I had a lot more interaction with health visitors and they would have like open sessions where you could go along and talk to somebody while your child plays*,* but I think because of the pandemic*,* things were very different with [my youngest child]*,* and it just wasn’t the same. […] I couldn’t see anyone face to face really.*

P2, Mother, English Language Focus Group.

HV/NNs recognised that these strains on resources were not solely caused by the pandemic but were multifactorial, and that this impacted on referrals and waiting times:

*Right now*,* with current situations*,* we make the referrals but due to whatever: our funding*,* COVID…*,* parents aren’t being seen in the estimated time. The speech and language time assessment: how long is that?*

HVNN2, Health Visitor.

Although the impacts of the COVID-19 Pandemic were recognised as important, HV/NNs also felt their service was uniquely positioned to continue to provide care in difficult conditions:

*Definitely since COVID – just the workload is just increasing; the needs*,* times 100. […] You can see that generation of children that have missed out. […] That’s why I feel like our job really comes into effect because we’re able to pick it up and support children and […] give them the best start.*

HVNN4, Health Visitor.

#### Subtheme 2.2: inflexibility

When exploring the accessibility of health visits, parents raised concerns about accessing healthcare services more broadly: they reported that it is hard to get appointments; they cannot get in touch easily; and appointments are often inconvenient. How these diverse parental responsibilities shape accessibility was also recognised by the HV/NNs:

*This time we had to take them for the two-year review. So obviously it became difficult to take them in with the other children. You have to take them and find childcare for them […]*.

P5, Mother, Sylheti Language Focus Group.

*I think it’s a cluster of a number of things. So*,* it could be parents working*,* child in nursery*,* other children to look after*,* a carer for someone… You know*,* so it’s a small percentage of everything that makes this huge*,* big bundle of things.*

HVNN5, Nursery Nurse.

This difficulty in accessing appointments highlighted the strain on primary care. Beyond an increased case load, unreliable availability of equipment, and the inability to have a set space to work from, these limited resources have taken flexibility away from both HV/NNs and parents.

*If you have a place*,* there is a scale*,* there’s everything there and you’re in the Children’s Centre*,* you can signpost to the Children’s Centre staff. And you can see more people. […] It’s like we’re losing the venue. I used to go to [Children’s Centre] during my reviews there. I’ve lost that room. I’ve lost the room in [a different Children’s Centre] so it’s getting really hard for you to see someone properly.*

HVNN6, Nursery Nurse.

An inflexible system also had the potential to impact parents differently depending on income and employment circumstances. Often the impact was larger on working mothers on lower incomes:

*Out of office hours would be good to work with parents because like*,* I mean*,* I’m quite lucky at this point that I have afternoons typically available. And I can sort of move stuff around if I need to because I’ve got my partner. But a lot of people are not that lucky. And then they have to take like time off. And I think especially if you’re on a low-income family*,* that might really prevent it from you going because they just can’t afford it.*

P1, Mother, English Language Focus Group.

#### Subtheme 2.3: lack of continuity of care resulted in parents receiving mixed advice

Both parents and HV/NNs expressed dissatisfaction at the lack of continuity of care; parents desired to see the same HV/NN who could be a point of contact throughout their child’s early years and found having to repeat information to different HV/NNs frustrating.

*Sometimes*,* we’ll have a problem and we don’t want to talk to everyone about it and repeat it many times. You just want to have one health visitor and we talk to them. That’s it. That’s easy. I know we had that before - they allocate one to you. But because of COVID we haven’t been able to get in touch with our regular health visitors.*

P5, Mother, Sylheti Language Focus Group.

Moreover, the benefits of having a single HV/NN included the feeling that they will understand changes more personally and be able to provide personalised support to a greater degree:

*The first thing I would like is to have one person; one point of contact*,* which we can rely on […] if you have someone who knows everything from the date of birth until five years*,* then you feel more comfortable to talk.*

P7, Mother, English Language Focus Group.

Parents’ frustration was compounded by instances of receiving mixed advice from different HV/NNs, while similar frustrations with mixed advice are shared by HV/NNs who found that they need to enforce new advice frequently:

*Some are more up to date with sort of latest research*,* latest advice*,* things that might have changed in sort of the last 10–20 years*,* and others less so. […] Like it’s very dependent on the individual*,* which is really dangerous if you don’t know the latest research yourself and you just trust whatever someone tells you.*

P1, Mother, English Language Focus Group.

*Because sometimes these parents may have*,* not just as first-time parents*,* they could have other children. And over the years*,* we can all agree that health visits have changed with regards to the information we give parents*,* because by the time they’ve had their first child*,* then they’ve had another child*,* it’s all changed. So obviously*,* for example*,* like for weaning*,* now we say it’s six months*,* whereas before*,* it used to be three months.*

HVNN1, Nursery Nurse.

#### Subtheme 2.4: power to refer for health and social issues

The disempowerment of HV/NNs was evidenced in the inability to make direct referrals, and the lack of services to refer to, alongside the limited capacity to be flexible with appointment times and locations:

*If this is the system that’s supposed to stand in for [greater access to doctors] then kind of like empower this system more. If this is supposed to be what’s checking and kind of doing the more regular check-ups and our medical interface with you know*,* children’s specific staff*,* then this maybe needs to be kind of like empowered more to make referrals to you know*,* do whatever it is that needs to be done.*

P3, Mother, English Language Focus Group.

The HV/NNs shared in this frustration regarding the inability to refer: “*Even if we wanted to refer to like the obesity clinic as clinicians*,* we can’t.”* (HVNN4, Health Visitor). Wider systemic limitations on the capacity of the NHS and its responsiveness to children’s needs were implicated in preventing health improvement and in reproducing parental anxieties:

*We recognise the issue*,* and then we don’t have any services that we can signpost to*,* or there’s no sort of clear pathway of what to do when the issue is recognised. And actually*,* that can raise anxieties further with families*,* because you’ve sort of left them*,* haven’t you? You’ve identified a problem. You’ve let them know what the issue is*,* and then you’re not doing anything about it.*

HVNN7, Health Visitor.

Parents also showed feelings of disempowerment, for example in the limited ability to advocate for themselves and the reliance upon professionals with strained resources to identify and address needs. Parents suggested that HV/NNs may be well positioned to help in contexts where accessing support is particularly difficult:

*They could ask about the house. If the conditions aren’t right*,* they could guide us towards finding the right support from the council and link us up to them. They look into different issues but there remains a major problem. For whoever’s very needy*,* they could speak up for them […] Like they need to understand that if it’s overcrowded*,* for example*,* obviously that affects the growth of the children.*

P8, Father, Sylheti Language Focus Group.

### Theme 3: desiring a preventative model

Parents and HV/NNs desired a focus on prevention which would be beneficial to children and families, as well as the healthcare system. They pointed out how prevention is already part of the system but could be maximised, and ways in which more prevention, specifically screening, would be desirable.

*Measurements can pick up signs*,* you know*,* evidence when things are going wrong. We know that development and thriving might be affected by things that are going on at home. […] What’s this child experiencing that might be meaning that they’re not eating*,* and they’re not growing?*

HVNN3, Health Visitor

*I think in other countries they definitely do like regular tests*,* or like a blood test at such and such age where you know*,* things might be picked up that you might not see until much much later*,* like actual physical outward symptoms […] Like let’s check actually like his tummy or this and that […] Can we just check like that everything is right?*

P1, Mother, English Language Focus Group.

However, there was the recognition that a more preventative model requires upfront resources, which were seen as lacking in the current health visiting system.

*Check-ups aren’t happening. […] it’s what you said: preventative. You’re saying check-ups and it doesn’t feel like there are check-ups.*

P3, Mother, English Language Focus Group

*One of the biggest things*,* I think*,* is resources as well. You can’t be inviting children every two to three months*,* unless you’ve got an identified need*,* because sometimes clinic spaces become an issue. And those spaces need to be reserved for families where there is an actual need. So you can’t take a blanket approach to all the children that are under your sort of care.*

HVNN7, Health Visitor.

## Discussion

### Summary

We sought views on the Healthy Child Programme from health visitors, nursery nurses and parents, and generated three interconnected overarching themes: the role of the HV/NN and the health visiting service; strained staffing and resources; and access to holistic care. There was agreement between parents and HV/NNs that there is a general lack of understanding of what the role of the HV/NNs is, and the importance of the service. Each group felt that this lack of clarity prevented optimal use of the service. Groups also agreed there were limitations on the service posed by the lack of resources available, including low staffing levels and lack of onward resources for families and children with additional needs across social and biological contexts. All participants expressed a desire to empower the health visiting service to deliver preventative and holistic care. While parents wished for more frequent and consistent contact with HV/NNs, they were realistic about the challenges posed by the current lack of resources.

### Strengths and limitations

This study has several strengths; by facilitating parental discussions in both English and Sylheti, we were able to include perspectives from under-represented parents. We were also able to simultaneously capture data from parents and health care providers, to obtain a rounded picture of the challenges and opportunities of the health visiting service. The use of focus groups allowed us to collect data on a range of perspectives with limited resources. The wider project from which these data were generated is concerned with quantitative aspects of the 2-year health and development assessment, and these qualitative data add depth to the overall picture of the service.

This study also has limitations; the number of participants was small and self-selected. However, we do not consider the small sample size to be a significant limitation, as we did not seek to reach ‘data saturation’; RTA requires deep engagement with the data and reflexive interpretation [17], which was prioritized over data saturation. Although all parents participating in CDGEL during a timeframe were invited to the qualitative sub-study, parents needed to agree to participate in this optional extra focus group, making parents who are more motivated likely to be over-represented. Parents with multiple caring responsibilities or with financial or physical difficulties are potentially under-represented in our findings, although we provided travel costs and childcare in an effort to reduce these biases. Paternal perspectives were inadequately captured, as nearly all of our participants were mothers. As ethnicity data was not collected, the study was unable to draw on specific culturally and ethnically rooted factors influencing their experiences. Our findings relate to the health system in England, and therefore do not apply to other healthcare contexts.

### Comparison with existing literature

This analysis adds to the existing understanding of how parents feel about the health visiting service by also considering the perspective of the health care providers who deliver this service. Previous work has highlighted how parents appreciate feeling understood and listened to [6, 7]; our work generates some ideas as to how this could be achieved, by showing that both parents and HV/NNs acknowledge that the role of the health visiting service is not well understood. Uncertainty around the role of the HV has previously been found to negatively affect some mothers struggling with mental health difficulties in the postpartum period [19], further aggravated by a reported lack of clarity around referral pathways, in particular when assessing perinatal mental health [20]. It has been suggested that the role of the health visiting service is hard to define due to the broad scope of the service [21]. This lack of clarity was observed in all of our groups, and the impacts ranged from inadequate use of the service, to mistrust and misunderstanding between parents and HV/NNs.

The focus on resources in each group echoes previous reports and research on both the health visiting service and the health service more generally. Well-resourced public health interventions have strong evidence of being impactful and cost-effective [22]. Despite this, recent years have seen declining NHS resources, including in health visiting [9]. A recent report from the Academy of Medical Sciences recommended urgently improving the family and child health workforce (including health visitors) and reducing fragmentation across sectors, as well as involving the perspective of service users in service development [15]. We are not aware of other studies that have highlighted parental desire for increased childhood screening and contact with a HV/NN, but we note that the number of mandated visits in the English HCP is lower than those in the other devolved nations (equivalent programmes in Scotland, Wales and Northern Ireland mandate between eight and eleven visits between pregnancy and age 5 years [23].

### Implications for research and practice

This analysis raises various implications for research and practice. Health policy makers should consider the relationship between parents and HV/NNs as a key factor in ensuring the effectiveness of the programme. Managing parental expectations of the service by emphasising the role of the HV/NN and what the service can and cannot provide could promote understanding and adequate use of the service. Parental desire for continuity of care where possible would likely improve collaboration between service and users. Parents also desired more visits, at times which are more convenient. A return to health visiting, where HV/NNs attend families’ homes, could help attenuate inequalities in access to the service, although this would require increased funding. Special consideration of the needs of migrant families and their diverse expectations of the service, shaped by their experiences in their countries of origin, would also help bridge the gap between parental and healthcare professional expectations.

We also identified a need for increased empowerment of the health visiting service; HV/NN are one of few points of contact for vulnerable families, which has the potential ability to link to other healthcare and local authority services. In this way, an empowered health visiting service could act as a gateway to access other services, and to advocate for the needs of these families. However, the needs of parents and HV/NN need to be balanced, and resources also need to be made available to HV/NN if there is to be higher expectations of their role. However, this is difficult in the face of current lack of resources in the healthcare system more widely. A review of models for well child programmes, such as the HCP, suggests that integrative frameworks have strong evidence for achieving better child health outcomes [24]. Integration of different systems concerned with child wellbeing, such as health, education and social systems, as well as family-centred care which is achieved collaboratively alongside healthcare professionals are recommended.

Collaborative and participatory mixed methods research which encompasses other regions in England is needed to better understand the role of the health visiting service nationally, but also how it could be used optimally in ways that are welcome and acceptable to both families and HV/NNs.

### Electronic supplementary material

Below is the link to the electronic supplementary material.

**Supplementary Material 1:** Health Visitor focus group interview guide

**Supplementary Material 2:** Parent focus group interview guide

## Data Availability

The data analysed as part of this qualitative study are available from the corresponding author upon reasonable request.

## Abbreviations

HCP: Healthy Child Programme
HV: Health Visitor
NN: Nursery Nurse
NHS: National Health Service
CDGEL: Child Development and Growth in East London study
RTA: Reflexive Thematic Analysis
P#: Parent participant number
HVNN#: Health Visitor or Nursery Nurse participant number
