# Supplementary material for: Parental and healthcare provider attitudes towards the Healthy Child Programme in England: a qualitative analysis
Source: BMC Public Health. 2024 Aug 28;24:2342. doi: 10.1186/s12889-024-19515-5 (PMC11360489; doi:10.1186/s12889-024-19515-5)
Supplement: Supplementary file 1 — Supplementary Material 1: Health Visitor focus group interview guide [file 12889_2024_19515_MOESM1_ESM.docx]

**Focus Group Discussion Guide**

**V.2.0 AUGUST 2022**

**Instructions for moderators:**

- Please try to create a welcoming and open environment where participants feel safe to share, including negative opinions or experiences.
- Agree on ‘ground rules’ with the group, such as confidentiality, engagement, respecting views of others, and practical issues such as switching off mobile phones (see script below).
- Please prompt participants to expand on their answers. If appropriate, take notes and follow up after the participant or group has finished speaking.
- Follow ups/prompts:
  - *Maria, you mentioned …. Can you tell us some more about that? Has something like this happened to anyone else?*
  - *Some of you mentioned that …. Could you say some more about that?*

| Topic | Questions / Script |
| --- | --- |
| **Welcome and introduction** | *Welcome to this session and thank you for being here. My name is …, and this is … we are … from....*  *You were invited to participate in this session because you are currently a Health Visitor or Nursery Nurse working within the Healthy Child Programme and providing routine healthy child check-ups. We are hoping that your thoughts and ideas could help improve the service for parents and children, and your experience as providers. In the long term, we hope results from this study will help improve health outcomes for children across England.*  *We are currently trialling the use of a growth screening algorithm which uses a child’s height and their parents’ heights, as well as past height measurements, to see if they are growing appropriately. We would also like to hear your opinions on the use of these kinds of tools within your standard practice.*  *There are no right or wrong answers, only different points of view. Please feel free to share positive and negative experiences and opinions, as they are both really important to our understanding.*  *We will be recording the session. This is to help us make sure we don’t miss any of your comments, as we probably won’t be able to make notes on everything that is said fast enough. We will be transcribing these recordings, and your names will not be used in any of the reports. All your contributions will be completely anonymous. We also ask that you speak one at a time as this makes the recording easier to understand.*  Housekeeping [to be prepared before session according to the session setting]:   - - Fire alarms, fire exits   - Feel free to keep phones on, but if you have to answer a phone call please do so outside   - Refreshments   Group introduction:  *So now I will ask you each to introduce yourselves by giving your name, where you are based, and tell us what you think the best, and hardest, things about working with this age group.*   - - Wait five seconds for volunteer   - If no volunteer – nominate a group member who seems outgoing to start |
| **Experiences of working in the healthy child programme** | *Do you think the Healthy Child Programme is achieving its goal of improving the health and wellbeing of children?*  *What could the programme be doing better?*  **Prompt:** *Why is the programme important? What do you think the most important part of the programme is? Is this aspect of the programme overlooked?* |
| **Opinions on growth screening** | *What about measuring children’s height and weight – do you think that is an important part of the programme?*  *Are there any challenges associated with monitoring children’s growth?*  *How do you think children’s growth should be monitored?*  **Prompt:** How often should weight and height be checked? Is checking weight and height enough/not enough?  *What do you think should happen when children are identified as having problems with their growth?*  **Prompt:** *What do you do when you suspect a child is not growing well? What do you think are the implications for children who are not growing well?* |
| **Use of parental height and algorithm** | [Summary here of how the algorithm is used in practice]  OR  *I previously mentioned an algorithm we are trialling. The use of this kind of algorithm shouldn’t change your standard practice; however, sometimes it may be useful to collect some further information, for example, parental height.*  *Do you think there are any benefits to using automated systems like this to assess children’s growth?*  **Prompt:** *What about downsides to using an algorithm to help make decisions about whether to refer children for further clinical investigation?*  *What challenges do you think the service could face if incorporating a programme like this?*  *Do you think there would be an impact on how much time the visits would take?* |
| **Opinions on development screening** | *What actions do you take when you think there might be a problem with a child’s development?*  **Prompt:** *Do you think it’s important to screen children’s development?* |
| **Barriers to access** | *What could be done to make it easier for you to do your work?*  **Prompt:** *Do you think there are ways in which Health Visitors could be supported to provide the programme?*  *What do you think stops parents from taking their children to their check-ups?*  **Prompt:** *Do you think there are ways in which parents could be supported to participate in the programme?* |
| **Wrap up** | Moderator sums up discussion in four to six key points.  *Is there anything else that we have not talked about regarding growth monitoring or the Healthy Child Programme that you think is important?* |
