# Supplementary material for: Parental and healthcare provider attitudes towards the Healthy Child Programme in England: a qualitative analysis
Source: BMC Public Health. 2024 Aug 28;24:2342. doi: 10.1186/s12889-024-19515-5 (PMC11360489; doi:10.1186/s12889-024-19515-5)
Supplement: Supplementary file 2 — Supplementary Material 2: Parent focus group interview guide [file 12889_2024_19515_MOESM2_ESM.docx]

**Focus Group Discussion Guide**

**V.2.0 AUGUST 2022**

**Instructions for moderators:**

- Please try to create a welcoming and open environment where participants feel safe to share, including negative opinions or experiences.
- Agree on ‘ground rules’ with the group, such as confidentiality, engagement, respecting views of others, and practical issues such as switching off mobile phones (see script below).
- Please prompt participants to expand on their answers. If appropriate, take notes and follow up after the participant or group has finished speaking.
- Follow ups/prompts:
  - *Maria, you mentioned that…. Can you tell us some more about that? Has something like this happened to anyone else?*
  - *Some of you mentioned that … Could you say some more about that?*

| Topic | Questions / Script |
| --- | --- |
| **Welcome and introduction** | *Welcome to this session and thank you for being here. My name is …, and this is … we are researchers Queen Mary University of London.*  *You were invited to participate in this session because you are a parent or carer of a child who recently had an appointment with the health visitor where their height, weight and development were checked. We are interested in hearing your opinions and experiences of these types of routine health care visits for your child. We are hoping that your thoughts and ideas could help improve the service for parents and children, and in the long-term help improve health outcomes for children across England.*  *There are no right or wrong answers, only different points of view. Please feel free to share positive and negative experiences and opinions, as they are both really important to our understanding.*  *We will be recording the session. This is to help us make sure we don’t miss any of your comments, as we probably won’t be able to make notes on everything that is said fast enough. We will be transcribing these recordings. Your names will not be used in any of the reports. All your contributions will be completely anonymous. We also ask that you speak one at a time as this makes the recording easier to understand.*  Housekeeping [to be prepared before session according to the session setting]:   - - Fire alarms, fire exits   - Feel free to keep phones on, but put them on silent if possible, and if you have to answer a phone call please do so outside   - Refreshments   Group introduction:  *So now I will ask you each to introduce yourselves by giving your name, the name of your child, and tell us what you think the best, and hardestt, things about having a two year old child are.*   - - Wait five seconds for volunteer   - If no volunteer – nominate a group member who seems outgoing to start |
| **Experiences of accessing healthcare** | *How do you feel about your child getting regular check-ups from GPs or HVs?*  **Prompt:** Do you think your regular check ups are too often? Not often enough?  *What do you think should happen when children go for their check-ups?*  **Prompt:** *For example, thinking about your child’s last visit, was there anything you* ***wish*** *the Health Visitor had checked or asked you about? Or was there anything in the visit that was unnecessary, in your opinion?* |
| **Opinions on growth screening** | *Have you ever had any concerns about your child’s growth?*  **Prompt:** *Is your child’s growth something that you think about as a parent?*  *And how do you feel about your child’s weight and height being measured?*  **Prompt**: *Are you happy for your child’s growth to be routinely monitored?*  *Why/why not?*  *How easy have you found having your child’s height measured in the past? Are there places you can go to do this, or have you ever asked to have your child’s height measured?*  *Do you think measuring height at your child’s visits adds too much burden onto parents?*  *As part of your participation in this study you were asked to have your height measured or report your height. Do you think parent’s heights should be taken into consideration when checking a child’s growth? Why/why not?*  *As part of your participation in this study, you were offered access to an app to monitor your child’s growth. Did anyone in the group use this app? How was your experience?*  **Prompt**: *Would you be interested in using something like this?*  *What would make using an app like this easier?*  *What was the reasoning for not wanting to use the app?*  *What do you think should happen when there are concerns about a child’s growth?* |
| **Opinions on child development screening** | *How do you feel about the health visitor asking you about your child’s development?*  **For clarity:** By development we mean how well they are reaching milestones, their speech, movement and social skills.  **Prompt**: *Have you ever had any concerns about your child’s development?*  *What do you think should happen when there are concerns about a child’s development?*  *Do you think there is a link between how a child grows and how well they are developing?* |
| **Barriers to access** | *What might stop parents from taking their children in to their appointments?*  **Prompt:** *Have you taken your child to all the GP and HV appointments you have been offered?*  *What could be done to make it easier for you to access all the routine appointments for your child?*  *How do you feel about referrals for further investigation or care being made as a result of the HV visit?* |
| **Wrap up** | Moderator sums up discussion in four to six key points.  *Is there anything else that we have not talked about regarding child check-ups or growth that you think is important?* |
